# A Victorian nature cure philosophy as a reconciliation of Romantic Naturalism and laboratory medicine: the case of E.W. Lane’s (1823–89) hygienic medicine

**DOI:** 10.1017/mdh.2024.42

**Published:** 2025-01

**Authors:** Min Bae

**Affiliations:** Busan University of Foreign Studies, Busan, South Korea

**Keywords:** Nature Cure, Romantic Naturalism, Laboratory Medicine, Vitalism, Laws of Health, Hygienic Medicine

## Abstract

This article primarily concentrates on the theoretical and intellectual dimensions of nature cure, particularly efforts to revive it during the second half of the nineteenth century. Little is known about specific medical professionals or theories concerning the Victorian philosophy of nature cure, although this philosophy is mentioned in general terms in studies on alternative medicine and hygiene. This article illuminates a Victorian nature cure philosophy through the works of Edward W. Lane (1823–1889). As a physician and hydropathist, Lane aspired to create a new form of medical system, ‘hygienic medicine’, seeking answers to the questions ‘What is disease?’ and ‘What is medicine?’ throughout his career. Lane was among many physicians interested in nature’s healing power during his time. However, few undertook as thorough a theorisation of nature cure philosophy as Lane did in the latter half of the nineteenth century, a period that presented unprecedented challenges in reconciling medicine with nature. This study explores the subtle nuances of the concepts that Lane actively used in his theoretical explanations, including ‘nature’, ‘laws of health’ and ‘vital force’, interpreting his efforts as a reconciliation between Romantic naturalism and laboratory medicine. The aim of this study is not to re-evaluate the significance of Lane’s medical theory itself. It rather uses Lane as a lens to reveal the intricacies of Victorian nature cure philosophy.

## Introduction

In a 1952 *BMJ* article, a doctor poignantly remarked, ‘In any case, my colleagues will agree with me that so far as the cure of diseases is concerned, we entirely depend on Nature. The labels “Nature-Cure” and “Naturopathy”, therefore, are apt to mislead the public’. Contrasting such a view, medical historian Richard Shryock wrote in 1931 that ‘Personal hygiene was an old, old story, but carrying it to the people with the fervor of a crusader was something relatively new’.^1^

The ‘nature cure’ philosophy has long been an extremely ambiguous concept, not least because of its chequered history of embracing a wide range of therapeutic and philosophical elements. The popular ‘holistic health movement’ in the late twentieth century, which featured a broad array of alternative medical therapies, sharing the notion of nature cures,^2^ was a continuation of the eclectic healing practices from the previous century.^3^ Although reaching any consensus on the definition of ‘nature cure philosophy’ seems unlikely, this study analyses what might be considered one of the clearest forms of Victorian nature cure philosophy, as presented by Edward Wickstead Lane (1823–89).

During his career, Lane persistently engaged with mainstream medical discourse despite his unconventional theory, which was well presented in his major work, *Hydropathy: or, Hygienic Medicine* (1859, hereinafter *Hygienic Medicine*). This work, as a reviewer described, ‘acknowledges openly the supreme power of nature…giving her fair-play in performing her cures,’^4^ while redefining hydropathy as a ‘methodised system of hygiene – for it is nothing else’.^5^ He ultimately sought to explain the philosophical relationship between medicine and nature. This exploration continued in subsequent works, through ‘What is Disease?’ (1874) and ‘What is Medicine?’ (1875) to *Hygienic Medicine: The Teaching of Physiology and Common-Sense* (1885), until the last decade of his life.

In the latter half of the nineteenth century, during which ‘the language of medicine became modernised into terms we use today,’^6^ few of Lane’s contemporaries were as committed to theorising and promoting the philosophy of nature cure in medical discourse. This hesitation, mainly stemming from the declining favour of deductive views on disease and medicine among medical professionals, was compounded by growing anxieties over societal challenges to their authority, as vividly expressed in a denunciation of the prevalent ‘infatuation’ – a ‘mixture of the most unreasonable medical scepticism with the most infantile superstition, the same intelligence…which believes nothing because it believes all’.^7^

The initial questions were: What were the core philosophical and cultural elements that drove Lane to consistently promote his dissenting medical views against dominant perspectives, and what strategies did he employ to advance his philosophy at the risk of being misconstrued as a medical sceptic or criticised as unprofessional? Placing Lane’s perspectives on disease and medicine within a historical current that may be termed Victorian nature cure philosophy, I interpret his theoretic work as an endeavour to reconcile Romantic Naturalism with laboratory medicine, while exploring the key concepts, including ‘nature’, ‘laws of health’, and ‘vital force’ in nuanced detail.

The aim of this study is not to re-evaluate the significance of Lane’s medical theory itself. His medical ideas were not highly innovative in the Whiggish sense, and his works, which failed to achieve medical and social recognition, might seem today like an incondite miscellany of medical philosophy and knowledge. I do not dispute such a characterisation. However, a good number of incondite miscellanies were published by hydropathists, especially in the 1840s. Compared with those earlier works, Lane’s works demonstrate a clear philosophical and theoretical direction, distinctly towards nature cure, beyond mere cold-water therapy. In this respect, this study, using Lane as a lens, aims to illuminate the widening gap between the nascent state of modern biomedicine and the medical undercurrent of nature cures during their most challenging period of coexistence within Victorian medicine. Exploring this gap can deepen our historical understanding of the intrinsic tensions in medicine’s relation to nature.

Before examining the historiography of nature cure philosophy, a brief introduction of the protagonist of this essay may be necessary. Lane, an Edinburgh-trained physician and hydropathist, was born in Quebec in 1823 and raised in Montreal. After his mother died in 1832, he moved to Edinburgh,^8^ where he studied law at the University of Edinburgh (receiving his MA in 1844). After becoming an advocate and marrying, he shifted to medicine, and upon graduation (from Edinburgh with his MD in 1853) he opened a hydropathic establishment in Moor Park, Surrey (1854–59), where he articulated his medical theory under the name of hygienic medicine, while treating notable patients such as Charles Darwin and George Combe. In 1860, Lane moved to Sudbrook Park, Petersham, and practised until 1879 before relocating to Harley Street, London, where he remained listed in the medical directory until his death in 1889.

## An untrodden path to Victorian nature cure philosophy

Victorian advancements in nature cures, distinct from the Georgian versions represented by John Wesley (1708–91) and William Buchan (1729–1805), have predominantly been studied regarding their cultural or social manifestations rather than their theoretical and intellectual dimensions. Victorian nature cure philosophy has been approached mostly through the medicalisation of hygiene and the proliferation of heterodox healing systems.

Hygiene embodied nature cure philosophy in the first half of the nineteenth century. Its medicalisation, though not a new phenomenon, developed throughout the nineteenth century often as part of health reform movements but also due to commercial needs and the professionalisation of medical disciplines. Andrew Wear, Logie Barrow and Virginia Smith highlight the holistic nature of early nineteenth-century hygiene, with the 1840s marking the peak in the popularity of neo-Hippocratism in Britain, influenced by European hygiene movements.^9^ Until the mid-century, elite orthodox physicians, most notably John Forbes (1787–1861) and James Clark (1788–1870), often sympathised with nature cures. A central figure in this historical context was Andrew Combe (1797–1847), who led ‘popular physiology’ in 1840s Edinburgh, an early Victorian update of the Georgian self-help tradition.^10^ The popularisation of personal hygiene, leading to social sensitiveness on water and air and an evangelical pursuit of exercise, accelerated its medicalisation. Vladimir Jankovic, discussing the evolving trend of Victorian ‘change of air’, notes: ‘As nineteenth-century climatotherapists gained more authority, discipline increasingly began to set the tone of medical expatriation’.^11^ Min Bae portrays Lane’s and Thomas R. Allinson’s (1858–1918) ‘hygienic medicine’ in terms of the relationship between the medicalising trend and the shifting concept of hygiene during the late century.^12^

Regarding heterodox healing systems, hydropathy, particularly Silesian peasant Vincent Priessnitz’s (1799–1851) therapeutic method, is considered a quintessential example of nineteenth-century nature cure culture. Its widespread adoption is rooted in the longstanding recognition of water as a natural healing agent, aligning with traditional regimen-based therapies. Robin Price regards hydropathy as key in the long history of ‘the doctrine of the natural healing processes’,^13^ noting its loss of ‘the return to Nature’ spirit as a decline of hydropathy as a medical heresy.^14^ Contrarily, James Bradley and Marguerite Dupree highlight hydropathy’s ‘dual existence’,^15^ indicating that initially a medical heresy, hydropathy began losing its therapeutic tension with orthodoxy in the 1860s.^16^ Other studies highlight nature-cure culture’s late-century revival, with a focus on naturopathy.^17^ P.S. Brown links Lane’s and Allinson’s hygienic medicine with naturopathy’s emergence, while remaining sceptical of a direct connection between hydropathy and naturopathy due to hydropathy’s waning influence among British doctors.^18^ In comparison, Smith more closely associates empiric healers of the era, including lay hydropathists, with the rise of naturopathy through the ‘Eclectic Movement’.^19^ Jane Adams views hydropathy’s naturopathic principles as foundational to health reform movements in the twentieth century, including naturopathy.^20^ In Brown’s and Adams’s studies, which solidify the presence of Victorian nature cure philosophy, Thomas L. Nichols (1815–1901, MD at New York University in 1850, immigrated to Britain in 1861), who significantly influenced Allinson, is illuminated as a theoretical bridge between hydropathy and naturopathy in Britain. In comparison to Nichols, who reflected North American social and spiritual radicalism rather than the British medical profession’s theoretical diversity, Lane’s perspective on nature cure more accurately unveils a concrete web of ideas within the Victorian medical minds, although, unlike Nichols, he did not have any direct contact with later naturopathy.

Given the above historiographical contexts, reducing Lane merely to hygiene or heterodox healing contexts may obscure a deeper understanding of his and other Victorians’ nature cure philosophies. Lane’s theory is a creation of his time but a subtle departure from the conventional medical views of regimens, drugs, and water embraced by physicians attuned to the *vis medicatrix naturae* (the healing power of nature). Victorian physicians acknowledged nature’s healing role, but in treating their patients, they prioritised intervention over waiting for nature to effect a cure. Especially towards the late century, social expectations to combat infectious diseases drove many elite physicians to develop laboratory medicine and cell theories, leading to ‘a rash of claims for the role of organisms’ in diseases among medical professionals.^21^

This contrasted with the nature-cure culture among the public, which was deeply associated with the medical market, as reflected by the popularity of hydropathy. James Whorton’s and Susn Cayleff’s studies show Russell T. Trall’s (1812–77, MD) incomparable significance to Lane in the history of hygiene and naturopathy, attributable to the American medical market, which was more receptive to nature cures and heterodox practices.^22^ Like Lane, Trall was interested in systematising and theorising nature cures and attempted to transform hydropathy into a more comprehensive healing system termed hygienic medication or hygeiotherapy. However, unlike Lane, his eclectic approach – incorporating vegetarianism and phrenology, running a health food shop and leading anti-tobacco campaigns – had broad public appeal. Additionally, unlike British homeopathy, whose vogue relied heavily on aristocratic patronage, the popularity of American hydropathy owed more to the medical market influenced by the liberal ethos of Jacksonian democracy.^23^

Compared with Trall in America, Lane in Britain focused more on theoretical and philosophical aspects of nature cures, striving for recognition in professional discourse rather than catering directly to popular medical market needs or joining social movements. Few studies, such as those by Brown and Bae, have focused on the aspect of nature cure within Lane’s intellectual journey.^24^ Conversely, his life as a lawyer-turned-physician operating a prestigious hydropathic establishment in a metropolitan suburb has been featured in many studies, particularly concerning the intellectual milieu at Lane’s Moor Park establishment (1854–59)^25^ and his involvement in the historically well-known 1857–58 divorce case.^26^ These studies often highlight his marriage into the Drysdale family, which helped him gain high social recognition early in his medical career. This asymmetry in the historiographical literature on Lane repeats, in a sense, the inclination towards social and cultural approaches to nature cures. Indeed, in terms of success in the medical market, Lane was no match for contemporary lay hydropaths such as John Smedley (1803–74).

From a philosophical and theoretical perspective, although Lane was not a Romantic-era physician, his lifelong intellectual exploration of ‘What is medicine?’ and ‘What is disease?’ resembled the introspective questions posed by Romantic thinkers. Considering that many historical studies recognise holism and vitalism as central to the philosophy of nature cure,^27^ it is unsurprising that, as Whorton notes, ‘trust in the body’s natural restorative powers was reinforced by the appeals of Romantic philosophers and poets to return to nature as the source of all truth and beauty’.^28^ However, few studies have explored the intricate historical and philosophical interconnections between lingering Romantic influences and emerging naturalist paradigms among Victorian medical professionals. Particularly, the specific relationship of Romantic medicine with naturopathic medical systems, such as hydropathy, remains less scrutinised in British history, unlike the clear ties between homeopathy and Romanticism in German history.^29^

In this regard, Romantic naturalism is key to understanding Lane’s and Victorian nature cure philosophy. Roderick Buchanan and James Bradley relate Robert Chambers’s (1802–71) *Vestiges of the Natural History of Creation* (1844, simply referred to as *Vestiges*) to ‘a romantic naturalism that all but removed God’s hand from daily affairs’.^30^ The radical philosophical views in *Vestiges* were controversial among both theologians and early scientists.^31^ Although rarely found in the history of science, the concept ‘romantic naturalism’ is liberally used in literary studies, often embodying ‘a tension between the real and the surreal, the materially tangible and yet mysterious and terrifying’.^32^ In comparison, in the history of science, Romanticism and scientific naturalism are often analysed separately, mostly regarding the influences of the former on the latter.^33^ This study, although it segments Lane’s medical theory into its Romantic and naturalist components to some degree, primarily examines the inherent tensions within his philosophy, particularly between medicine and nature, to capture his holistic perspective.

## Life as a physician hydropath: Beyond the ordinary unorthodox

Lane is best known as a hydropathist to Darwin and inspired a hydropath protagonist in Dinah Craik’s novel when she stayed at Moor Park.^34^ However, similar to his medical position as a physician – born in Canada and educated in Edinburgh – outside the metropolitan elite, Lane’s life diverged from the typical hydropathists’ of the era.

Lane began practicing hydropathy just after his graduation in 1853, unlike most first-generation hydropathists who medically qualified during 1825–40. Compared with them, whose leading figures mostly retired by the early 1870s,^35^ Lane, intellectually vibrant in the 1860s (he was 38 years old in 1861), continued to develop his unconventional perspectives on disease and medicine, as his 1870s publications illustrate. His early exposure to the tensions between hydropathy and orthodox medicine, notably around the Medical Act of 1858, also cemented his commitment to the unorthodox medical philosophy that persisted until his final years, unlike later hydropaths who began to practise in the 1860s and afterwards.^36^

At a more fundamental level, Lane’s deviation from mainstream medicine and hydropathy was driven by earlier life experiences that anchored his mind in Romantic and naturalist contexts. In 1847, the year when Lane was admitted to the Faculty of Advocates in Edinburgh,^37^ he married Margaret M. Drysdale, the eldest daughter of the late Sir William Drysdale (1781–1843), a former treasurer of Edinburgh and influential advocate of sanitary reform. Lane had long been acquainted with George Drysdale (1824–1904), Margaret’s younger brother, who reportedly drowned during a walking tour with him. Soon after marriage, Lane and his wife, accompanied by his mother-in-law, Elizabeth Drysdale, went to Dublin, where George, having returned alive, was studying medicine. After this trip, Lane began medical studies in 1849 with George, who returned to Edinburgh. They both had a keen academic interest in physiology, fuelled by the burgeoning interest in popular science in Edinburgh.^38^

George Drysdale significantly influenced Lane to adopt dissenting views on orthodox medicine’s understanding of the body and disease.^39^ Whilst Lane was undertaking his legal training, Drysdale was deeply encumbered with the matter of onanism. While both were in medical school, Drysdale wrote *Physical, Sexual, and Natural Religion* (1854), devotion to which led to his graduation being delayed until 1855. Published anonymously and later retitled *Elements of Social Science*, this work represented a Victorian moral undercurrent based on the intellectual tradition of free love, associated with radicals such as Robert Owen. Opposing the medical profession’s increasing emphasis on respectability and condemnation of sexual indulgence, Drysdale regarded it as immoral to encourage undermining health by suppressing natural drives. He aimed to revive strands of free-love ideals, which peaked in the Romantic era: ‘sexual disappointments and anxieties darken the whole sexual atmosphere, and have fostered the puritanism, which has of late years increased among us’.^40^

In a similar vein, Lane was philosophically inclined towards medical undercurrents based on the intellectual tradition of nature cure, opposing what he perceived as dominant unnatural trends. Lane shared George’s enthusiasm for reforming medicine and creating an enlightened medical profession,^41^ which was later reflected in *Hygienic Medicine*: ‘when the clear and broad doctrines of hygienic medicine have once fairly been taken hold of by the public at large, woe to the practitioner who fails to give them their due weight in the rationale of his medical treatment’.^42^ Lane, as J.M. Benn described, was one of George Drysdale’s ‘first converts to a physical, sexual and natural religion’, albeit less fervently than his younger brother, Charles Drysdale.^43^

Lane entered medical school during the era of ‘the collapse of traditional medicine’.^44^ Orthodox medicine was a target of criticism, as evidenced by a sensational article (1 September 1849) highlighting lower death rates under homeopathy in *Chambers’s Edinburgh Journal.* ^45^ Conversely, Henderson (1810–72), a homeopathy-supporting pathology professor, became embroiled in controversy with peers, including James Yong Simpson and Robert Christison, in 1851, leading to his expulsion from the Edinburgh Medico-Chirurgical Society and his position in the Edinburgh Royal Infirmary.^46^ In this confusing atmosphere, Lane found inspiration in John Hughes Bennett (1812–75), attending Clinical Medicine,^47^ a course renowned for teaching excellence.^48^ Bennett’s emphasis on molecular investigation using microscopy in physiology and pathology profoundly impacted Lane’s theoretical understanding of blood physiology as articulated in *Hygienic Medicine.* ^49^ Military surgery professor Georgy Ballingall (1780–1855) also influenced Lane’s enthusiasm for hospital reform^50^ – a theme prominently featured in his graduation thesis, which was dedicated to Ballingall.^51^

Despite his personal ties to homeopathy,^52^ Lane leaned towards hydropathy, which, during the 1840s, quickly gained popularity among intellectuals.^53^ In May 1854, Lane personally realised his vision for hospital reform by establishing Moor Park Medical and Hydropathic Establishment.^54^ The Drysdale family, particularly Elizabeth,^55^ helped draw intellectuals like George Combe,^56^ Robert Chambers,^57^ and Charles Darwin to Moor Park, which became a hub for medical and scientific discourse during their work on notable publications, such as Combe’s *On the Relation between Science and Religion* (1857) and Darwin’s *The Origin of Species* (1859). Surrounded by this intellectual atmosphere, Lane’s broader medical philosophy transcended the confines of hydropathy. George Combe (1788–1858) actively encouraged Lane to pursue a new system centring on ‘hygienic treatments’.^58^ In his book, Lane painstakingly explained ‘what Hydropathy is not,’ arguing that it was definitely ‘not the *Water-Cure*’.^59^ This effort was mirrored in a review of *Hydropathy: or, Hygienic Medicine*: ‘To merge, though it be only in the words of the title-page, the great science of hygienics in a single branch of therapeutics was a most unfortunate literary blunder’.^60^ The reviewer suggested that ‘Dr. Lane might without blame have named his book *Hygienic Medicine with special reference to Hydropathy*’.^61^ During the Sudbrook Park period (1860–79), he published *Old Medicine and New* (1873) and contributed medico-philosophical and public health articles primarily to *The Medical Press and Circular.* Despite advertising his establishment actively,^62^ he remained detached from medical or hydropathist networks. In his articles and his last work (1885), hydropathy was not even mentioned.

## Hygienic Medicine: a door to Lane’s medical philosophy

*Hygienic Medicine* is the revised edition of Lane’s initial work, *Hydropathy: or the Natural System of Medical Treatment* (1857). It encapsulated his faith in ‘*natural means of medical treatment*,’ comprising air, exercise, water, diet, and repose.^63^

The purported aim of the book was to present the ‘rational grounds of hygienic medicine’ and achieve ‘reconciliation between the practitioners of old physic and the more modern natural school’.^64^ Its preface identified its audience as ‘my professional brethren’,^65^ but both editions were advertised in a wide range of contemporary newspapers and magazines, well beyond the scope of the medical field.^66^ The book comprises six chapters. At a superficial level, by looking at the chapter titles, the book may appear to merely explain hydropathy. However, Lane describes its main problem in Chapter 1: an overreliance on the mechanical application of water. Chapter 2 delineates the theoretical ground on which, he believes, hydropathy should be practised and presents the main concepts of his medical philosophy. Chapter 3 is dedicated to his aetiological theory and Chapter 4 to his therapeutic principles for the systemic application of hygienic agents (natural agents). Chapter 5 expands on the concept of nature cure, and Chapter 6 discusses contemporary medical trends, highlighting the growing interest in hygienic philosophy.

*Hygienic Medicine* elicited mixed reviews from both professional and public media (at least twelve reviews across its two editions), garnering more attention than his *Old Medicine and New* (three or more reviews). Medical journals, including a notably harsh critique from *The Lancet* (22 August 1857), often disapproved, while general periodicals were more favourable, despite some criticisms from *The Athenaeum* (7 November 1857) and *John Bull and Britannia* (15 August 1857). The mixed feedback mainly arose from varying perspectives on hydropathy. Even *The Medical Times & Gazette* (8 August 1857), published by John Churchill, his book publisher, was unexceptional in its criticism of hydropathy.

Lane adhered to his theory of hygienic medicine presented in this book throughout his life. In ‘What is Disease?’ and ‘What is Medicine?’, he clarified disease as ‘a penalty of the broken laws of health’^67^ and defined medicine as the ‘Healing Art’, which included anything that ‘heals’, ‘whether it be a drug…or the return to the observance of the laws of health’.^68^ In his last work, he demonstrated his enduring commitment to his medical theory and revisited the fundamental questions about disease and medicine he had posed in the previous decade.

Orthodox medical doctors also endeavoured to reconcile hygiene and medicine, associating the notion of hygiene with the growing body of physiological knowledge.^69^ However, their understanding of hygiene was mostly confined to the preventive domain, aligning with sanitary efforts during the century’s public health movements.^70^ This contrasted with Lane’s view, in which if natural agents are ‘essential to the preservation of health’, it is equally true that ‘the very same physiological agents…are the not only safest but the surest means of curing disease’.^71^

Clearly, Lane’s medical thoughts bore similarities to those of many unorthodox practitioners. He shared their criticism of conventional medicine for ‘forcing nature to do what the doctor wishes’,^72^ a view widely supported, as seen in a review of his book: ‘drugs, in short, have fallen under general suspicion; and men revert, more or less practically, to an old idea…that there resides a curative power in Nature herself’.^73^ Lane also shared a typical Victorian moral sentiment, vocally championed by many of his contemporaries in evangelical tones, as exemplified by renowned Malvern hydropathist James Wilson (1807–67, MD): ‘Obedience to law, whether moral or material, natural or revealed law, is equally our duty and our interest. It leads to health of body and mind, to purity and peace. Disobedience, on the contrary, leads to sin and sorrow, to disease and unhappiness’.^74^ In terms of the nineteenth-century medicalisation of hygiene, both hydropathists and climatotherapists viewed their roles as more than merely prescribing water or travel: ‘it was a moral contract between doctor and patient’.^75^

However, Lane’s approach diverged from the prevailing hydropathic trend, which focused more on the mechanical and thermal aspects of applying water. This trend, over time, resulted in hydropathic institutions, similar to sanatoria for phthisis and private hospitals, increasingly demanding costly plans necessitating ‘the intervention of the capitalist’.^76^ As Adams notes, hydropathy was ‘incorporated into scientific medical hydrology from the last quarter of the nineteenth century,’^77^ The aspiration of ‘hydrotherapy’ for a ‘well-defined science’^78^ paralleled that of climatotherapy, which also ‘paid minute attention to the effects of different climates on the nervous and digestive systems’.^79^ For many practitioners advocating for prescribing hygienic agents, whether water or climate, the clinical role of specific agents was their main concern amidst the growing medical professionalisation.

In contrast, Lane was more interested in the holistic effects of natural agents’ comprehensive functions, while steering clear of technicalities: ‘natural agencies…when taken together, go to form a system, applicable singly or in conjunction with other means…and never entirely dissociated’.^80^ He critiqued contemporary hydropathy for ‘the great error…of substituting one of a group of interdependent curative agencies – water – as titular and absolute representative of the whole’.^81^ For a similar reason, he criticised other heterodox systems: ‘the Homeopathists in their practice have had the wisdom to lay much stress on hygienic considerations….But such things as exercise, air and diet…are with them regarded as only very ancillary…a fault which it shares with Old Physic’.^82^ Lane’s theory was aimed at ‘the natural’ system of medical treatment, as manifested by his original book title.^83^ This vision distanced him from both the ‘Water-Cure’ system, which was not particularly interested in the systematic use of hygienic agents and from the ‘old system of medical treatment’,^84^ which was reluctant to recognise fully the therapeutic usage of the agents sanctioned by nature.

## Romanticism in Lane’s nature cure philosophy

Regarding medicine, Romanticism is often associated with charismatic figures, such as Franz Anton Mesmer (1734–1815) and Samuel Hahnemann (1755–1843).^85^ Historians have long recognised that Romantic medicine, centring on vitalism and holism, inspired a resurgence of the traditional values of *vis medicatrix naturae* in many physicians’ minds.^86^

Lane viewed hygienic treatment as ‘exalting the *vis medicatrix*, and thereby placing Nature in the most favourable circumstances to cure herself’.^87^ However, this view needs to be distinguished from contemporary physicians’ focus on the ‘self-limiting nature of ailments’.^88^ David Skae, resident physician of the Royal Edinburgh Asylum (1846–72), advised his students that ‘it is astonishing what nature can do for the cure of disease – if she is left alone not interfered with by too much Doctoring. It is well known that all acute diseases tend towards a cure, exciting causes [and] sources of irritation being removed’.^89^

Compared with this interest in nature’s roles in the pathological healing process, Lane’s theory of hygienic medicine championed an idealised perception of nature as the wellspring of health. This medical perspective, reminiscent of the Romantic themes in William Wordsworth’s and Samuel Taylor Coleridge’s poems,^90^ was epitomised by Humphry Davy (1778–1829), one-time superintendent of Thomas Beddoes’s (1803–49) Pneumatic Institution: ‘Nature never deceives us. The rocks, the mountains, the streams, always speak the same language’.^91^ Lane’s belief that urban life hampered nature’s restorative efforts to balance one’s vitality also reflects the Romantic critique of industrialisation’s impact on human well-being, encapsulated in his reference to William Cowper’s line (from *The Task*), ‘God made the country, but man made the town’.^92^

This perception of nature – as the arbiter of health conditions – is closely associated with a comparative view of medicine and nature, a notion prevalent in the Romantic era. As Wordsworth’s writings poignantly illustrate, this perspective suggested that an absolute cure could not be expected to come by art alone, which could mostly achieve only alleviation.^93^ ‘Expectant medicine’, which Erwin Ackerknecht ascribed to the Paris Clinical School,^94^ and Max Neuburger related to the expanding knowledge of ‘the purposeful working of Nature’,^95^ was a major point of contention in Victorian medicine. It was advocated by physicians who insisted that ‘in many instances Nature unassisted can do everything’ but sometimes derisively described as ‘the art of laboriously doing nothing,’^96^ and even blamed for a ‘purposeful avoidance of intervention for the sake of pathological enthusiasm’.^97^

Although Lane acknowledged the constraints of medical practice, he actively sought to redefine its scope. Vigorously challenging the prevalent therapeutic scepticism in his time, he even critiqued John Forbes (1787–1861), who, alongside Bennett, was lauded as one of the ‘two high priests of Nature’.^98^

Agreeing, however…with the general tenor of Sir John Forbes’s ideas…as to the general efficacy of nature in working her own cure, I am not prepared to go all lengths with him as to the comparative inutility of art…. [T]his would manifestly be to confine the term [‘art’] within very narrow limits.^99^

Compared with Romantic medicine’s tendency to favour nature in the tension with medicine, Lane endeavoured to harmonise his robust rebuttal of medical scepticism with a Romantic reverence for nature. For him, nature responds to an unhealthy lifestyle with pain and disease, prompting individuals to adopt healthier habits. He viewed the role of doctors in nature cure as helping patients self-regulate their life habits by professionally organising the application of natural agencies, stating: ‘Its rationale is…that nature possesses within herself…her own means of restoration.… When her powers are not sufficient to this end…the aid…must be founded on a consideration of the primary laws of health as unfolded by physiology’.^100^ For Lane, this ‘aid’ meant supporting patients’ autonomous efforts for health rather than intervening in their helpless states dependent on doctors’ goodwill. This perspective did not diminish doctors’ roles but rather underscored their professional significance. In an article on the social role of medical professionals in public health, he likened them to clerics, suggesting they provide direct guidance to the poor on ‘how to live *physically*’ through home visits.^101^

Lane might also have seen the clinical implementation of hygiene as a lucrative medical niche. His hygienic hospital concept aimed to apply the ‘intensified degree’ of hygienic agents to patients under strict medical supervision. Lane’s ‘hydropathic sanatorium’,^102^ dubbed ‘a temple of hygiene’ in a review of his book,^103^ aimed beyond advanced water treatments or urban escape. It sought to create a space where ‘the patients mostly reside under the same roof with the physician, and eat at the same table,’ and ‘the greatest regularity is observed as to the times and ways of doing everything’.^104^

Lane’s firm belief in nature cures, underpinning this therapeutic communal setting and holistic lifestyle approach, was rooted in his Romantic vision of nature, transcending the pathological meaning of natural healing. As his relationship with George Drysdale implies, the lingering Romantic sentiments in the physiology of early 1850s Edinburgh significantly influenced Lane’s life. However, while showing a close affinity with Romantic medicine or contemporary hydropathy, the nature cure philosophy to which he remained committed throughout his life, requires a more nuanced understanding within the medical contexts of the second half of the nineteenth century.

## Lane’s naturalist view of the laws of health

While early nineteenth-century Europe saw a revived emphasis on empirical observation and an increasing recognition of pathophysiological concepts, these features were interrelated to the collapse of traditionalism.^105^ Simultaneously, increasing numbers of medical practitioners were attracted by the concept of natural laws – a concept expressed in Schelling’s dictum, ‘The higher perfection of the natural sciences…would be a complete intellectualisation of all natural laws, into the laws of observation and of thinking’.^106^ Particularly, the elevated awareness of the laws of health was associated with rising concern about hygiene among the lower-middle and working classes.^107^ In mid-nineteenth-century Britain, where therapeutic scepticism flourished, and the boundaries of medical orthodoxy were blurred,^108^ the laws of health, as a popular concept advocating self-care, thrived in the medical marketplace, as reflected in doctor-turned-author Samuel Smiles’ (1812–1904) *Self-Help* (1859).

The concept of the laws of health was not new to British medical practitioners. From a contemporary medical view, the philosophical principle of William Cullen’s (1710–90) system lay in ‘considering the human body as a congeries of animated organs, regulated by the laws, not of inanimate matter, but of life, and superintended by an immaterial principle, acting wisely, but necessarily, for the general health’.^109^ By the 1850s, many English physicians used the concept in a wide range of medical discourses with different focuses. Surgeon and medical officer of health Lionel J. Beale (1796–1871)^110^ stated that ‘all the laws of health may be reduced to the observance of cleanliness and temperance’.^111^ This succinctly reflected the prevailing sanitary and religious concerns related to the concept in Victorian society.

In comparison, Lane’s notion of the laws of health presented a more clinical and naturalist perspective. Although deeply influenced by Georgy Drysdale, Lane intended to align his medical theory more with the Combe brothers’ philosophical and medical discourses. George Combe’s phrenological view of human minds, which fell within ‘Romantic psychologies’, was rooted in the concept of natural laws, to which he argued Man is subject.^112^ The popularity of this concept led to vigilance among those who opposed the superiority of natural law over divine law, as seen later in the reaction to Robert Chambers’ anonymously published *Vestiges.* ^113^ Further developing the notion of natural laws in a medical context, Andrew Combe aimed to establish hygiene as ‘the principles or rules by which the highest health and efficiency of all our functions, moral, intellectual, and corporeal, may be most certainly secured, and by obedience to which we may, when once diseased, most speedily and safely regain our health’.^114^ Similarly, Lane argued that conforming to the laws of health meant a complete embrace and adaptation to natural agents: ‘Every living being, in order to be healthy, must have a sufficiency of nutritive, but plain, food and good water; he must enjoy pure air…. Nature has enjoined that these things shall be, if the human being is to flourish, and from her ordinance there is no possibility of an escape with health’.^115^

Sharing with the Combe brothers the concept of ‘natural laws’ and its teleomechanist basis, which posited the existence of lawlike principles that the body obeys,^116^ Lane detached the laws of health in his theory from the natural theology of the early nineteenth century. His perspective aligned closely with the Romantic ideal of ‘a pure, *disinterested* science’, independent of religious doctrine.^117^ In addition, his naturalist perspective echoed the rising Victorian professional elitism against traditional Anglicanism,^118^ as seen in his metaphorical plea for tolerance towards different medical theories: ‘Look at the Church…and reflect what the fate of even so small a section as the Established Church of England must have been, if…her policy had been to expel from her bosom every one of her children who was found dissenting’.^119^

Lane’s view of the laws of health, in this respect, was distinct from dominant contemporary medical views, which accommodated ‘theological concerns to sustain the notion of the corrupt and immaterial soul with a burgeoning medical science’.^120^ In *The Laws of Health* (1851), Beale viewed the term ‘laws of nature’ as being exploited by naturalists who attempted to evade the need for ‘our Creator’.^121^ For many hydropathists, too, spiritual well-being was considered indispensable for therapeutic purposes, and strict religious observance was incorporated into the hydropathic regimen.^122^

Although Lane used the same words as his era, such as ‘laws of health’ and ‘hydropathy’, his medical perspectives diverged from and were more dissenting than those of contemporary orthodox and unorthodox practitioners, both philosophically and clinically. He called contemporary therapeutic approaches the ‘art’ of medicine, not the ‘science’ of medicine,^123^ criticising their irrational reliance on drug medication. He also challenged the ‘sciences of yesterday’, dominated by ‘pathology and pathological chemistry’,^124^ arguing that the ‘philosophical foundation’ of medicine should rest on physiology rather than pathology for medicine to be truly ‘rational in its method’.^125^ As will be explored further, Lane’s prioritisation of physiology over pathology, along with his clinical and theoretical interpretations of vitality, holds special significance regarding his explanations of disease and medicine.

## What is disease?: Lane’s medical view of vitality

In *Hygienic Medicine*, Lane categorises diseases into two classes based on vitality: Class 1 with excess vital power and the more prevalent Class 2 with deficient vital power. Treatment involves either reducing or elevating ‘the economy’ to a ‘normal and healthy standard’.^126^ From an epistemological standpoint, this dualistic framework echoes the tradition of Scottish philosophical medicine, demonstrated by Cullen, who emphasised restoring bodily functions through stimulation or depletion,^127^ and William P. Alison (1790–1859), who classified diseases as febrile or nonfebrile, subdividing the former into inflammatory diseases and fevers proper.^128^

However, in terms of nosology and aetiology, Lane’s view of disease warrants a closer examination of its distinct features. Traditionally, diseases were regarded as states specific to individual patients rather than as independent entities, typically understood within a multifactorial causation framework. However, elaborate disease classifications since Thomas Sydenham (1624–89) led to an increasing recognition of disease entities, changing the ontological view of disease. In essence, Cullen’s nosology, which categorised a variety of fevers based on specific symptoms, was a simplified educational adaptation of Sydenham’s work. By the mid-nineteenth century, the doctrine of specificity became a dominant medical view of disease as ‘a pragmatic concept’ reinforced by quantitative pathological observations, endorsed even by lay practitioners, including homeopathists and botanists.^129^

Contrasting with this aetiological trend behind the evolving nosology, Lane’s aetiology centred on patient individuality, not disease specificity. Eschewing a focus on specific symptoms, Lane centred on the concept of disease as ‘the *nexus* that binds all diseases together’,^130^ positing that the fundamental cause of disease exists at the level of the whole. He sought a unified medical model that coherently defines disease and treatment, a feature typically seen in the medical philosophies of Romantic physicians such as John Brown (1735–88) and Thomas Beddoes.^131^ As Beddoes regarded the therapeutic principles for consumption as applicable to most other diseases, Lane stated: ‘The mass of chronic diseases are most effectually and most safely cured…by the identical means infinitely modified…according to circumstances, that are requisite for maintaining the animal economy in health’.^132^ This perspective needs to be distinguished from the quest for a ‘universal law’ in natural sciences. Contrary to the doctrine of specifics, Lane’s aetiology emphasised a personalised approach to disease, valuing the unique ‘circumstances’ of individual patients. His philosophy prioritised individual human experiences in medical treatment, akin to the Romantic philosophy of nature emphasising the ‘singular existence’ of all living organisms.^133^ Lane maintained his aetiology, even after acknowledging germ theory, as seen in his 1885 work: ‘Even as to these [infectious diseases], if we could examine narrowly and carefully enough, we should find that their ultimate cause must be derivable…from the violation of the same primitive laws of health’.^134^

Lane’s vitalist aetiology drew fundamentally from the Romantic concept of life, particularly Brunonianism. John Brown postulated that the principle of life, ‘excitement’ or the life force as the distinctive feature of life, applies equally to health and disease. Diverging from Cullen, Brown promoted a radical vitalist view within Scottish medicine that posited a single root cause for all disease manifestations, ascribing all diseases to either under- or overstimulation (asthenic or sthenic).^135^

In this respect, Lane’s view of disease is compared with the nature cure perspective of Russell T. Trall, who derided the Brunonian approach, stating that ‘the practice that naturally results from such a theory or phantasy is bleeding in one class of diseases, and brandy in the other’.^136^ Trall explained the fundamental cause of disease as ‘a *loss* of balance in the circulation and action of the various parts of the vital machinery’, attributed to ‘unphysiological voluntary habits’.^137^ While both Lane and Trall based their aetiology on vitalism and advocated way-of-life approaches, Trall’s aetiology was relatively more mechanical and functional, aligning with the prevailing notion of bodily purity, which focused on expelling morbid matter from the body, often combined with the conception of ‘inner rottenness’^138^: ‘From improper food, vitiated air, impure water, or suppressed perspiration, the blood may be loaded with morbific matters, which the vital powers are naturally disposed to expel through these depurating organs – the skin and kidneys’.^139^

While Trall’s criticism of Brown was somewhat erroneous,^140^ for early nineteenth-century physicians adhering to vitalism, bloodletting was a logical conclusion to sthenic (overexcitement) types of fevers, the *genus synocha* in Cullen’s nosology.^141^ Despite his aversion to such aggressive treatments, Lane’s aetiology was deeply anchored in Romantic vitalism. He considered a vital force to be embodied in blood, unlike Enlightenment vitalism’s functional approach.^142^ In Britain, Romanticism’s inception was closely tied to the substantive form of vitalism, as evidenced by John Hunter’s (1728–93) concept of the ‘life of blood’ in the discourse on the ‘vital principle’.^143^ Andrew Combe directly related the flow of blood to the level of vitality: ‘Great depression of mind thus leads naturally to imperfect respiration, a more sluggish flow of blood and the various diseases of diminished vitality, while great excitement induces full respiration, quickened circulation, and the various diseases of exalted vitality’.^144^ Echoing Combe, who was for Lane a philosophical bridge between Brown and him, Lane characterised pulmonary consumption (a Class 2 disease in his nosology) not just as a ‘constitutional disease’ but also as a ‘blood disease’.^145^ He argued that ‘It is to a certain depraved or altered condition of that vital fluid that the disease in all its forms is due’.^146^

Lane aimed to modernise Romantic vitalism by integrating microscopic discoveries. Lane’s view of physiology mirrored Johannes Peter Müller’s (1801–58) ‘data-driven *Naturphilosophie*’. Müller combined observation and experimentation, positioning physiology as an independent science while maintaining his vitalist perspective.^147^ In *Hygienic Medicine*, Lane sought to reconcile his radical vitalism, inspired by the Romantic concept of life, particularly its Brunonian version, with Justus von Liebig’s (1803–73) theory, which suggested a ‘molecular basis of continuity’ between organic and inorganic realms.^148^ Indeed, Liebig’s chemical physiology was adopted by many heterodox practitioners to justify their therapeutic regimes in the 1840s. Unlike most medically qualified hydropathists, who based their theoretical and philosophical frameworks on orthodox models,^149^ Lane ventured to theorise a new medical system, striving to create a framework that could effectively integrate Romantic vitalist aetiology with his naturalist perspective on the laws of health.

In these contexts of ‘blood disease’ and ‘molecular basis’, the central concept was the quality of blood, which Lane viewed as deeply associated with diet and digestive powers.^150^ His approach certainly bore a resemblance to modern humouralism, which resurged in the 1840s,^151^ led by figures like Carl von Rokitanski (1804–78). Rokitanski linked disease to an imbalance in the blood’s protein components, such as albumin and fibrin.^152^ Yet, Lane’s focus on vitality and microscopic vascular functions set his disease theory apart from Rokitanski’s chemically oriented hematohumoural theory, aligning it more closely with Charles J.B. Williams’ explanations, as Lane’s references to his *Principles of Medicine* (1843) suggest. Williams, a professor at University College London, posited that the red blood discs, which ‘appear to be the part of the blood on which its vivifying and calorific properties chiefly depend,’ could incite ‘a general excitement of the vital properties of the body’ when in excess.^153^ In this context, the most crucial and trusted key in Lane’s disease theory, in line with his efforts to reconcile his vitalist view with Liebig’s organic chemistry, was Bennett’s pathology. *Hygienic Medicine* illustrates Lane’s endeavour to interpret Bennett’s pathological insights through his own humouralist physiological explanations. In analysing pulmonary consumption, Lane described how ‘impoverished blood’ and its ‘low type of aplastic, albuminous, exudation’ tended ‘on the application of any exciting cause, such as cold,’ to ‘transude through the vessels into…the air vessels of the lungs’ with a ‘tendency to disintegration of tissue’.^154^

Lane’s theoretical formulation, aiming at reconciling the vitalist aetiological framework with microscopical knowledge, resembles what Rick Looijen terms ‘heterogeneous micro-reduction’ – a mix of ‘holistic research at the level of the whole and reductionist research at the level of the parts’, coupled with a ‘mixed strategy directed at the relations between the two levels’.^155^ Lane’s explanations on a micro level underpinned his overarching goal of elucidating principles governing the body as a whole. From today’s perspective, Lane’s aetiology can be interpreted as focusing on immunity over microbial agents, with ‘impoverished blood’ corresponding to blood in a weakened state of immunity.

This integration of vital force with pathohistological findings was essential in Lane’s effort to formulate a medical theory based on deductive reasoning. Clarifying the blood-disease connection was key to theorising the direct link between disease manifestation and the vitality principle. While Lane’s effort aimed to blend scientific research with philosophical reflection, characteristic of the Romantic era’s intellectual depth,^156^ it deviated from the new medical trend of his time, which sought to differentiate pathological from physiological knowledge, reconceptualising disease ‘as a set of processes occurring among cells and tissues’.^157^ On the other hand, the rapid waning of vitalism’s popularity in professional discourse contrasted starkly with the public’s robust enthusiasm for vitalist views of health and disease. While Trall mirrored the popular perceptions, such as bodily purity or inner hygiene, among the public, Lane’s Romantic vitalist elements, influenced by Andrew Combe, led him to make efforts – likely encouraged by his naturalist friends, at the Moor Park establishment – to rejuvenate the declining vitalist aetiology by crafting a nuanced theoretical response to new discoveries in laboratory medicine.

## What is medicine?: The relationship between disease and hygiene

As a logical extension of his aetiology, Lane advocated improving vitality through ways of life conforming to the laws of health. This direct link between vitality and hygiene reflected the Romantic influence. Beddoes envisioned hygiene as ‘a people’s science’, aspiring for health as a social ideal.^158^ Just as Beddoes regarded an urbanised commercial lifestyle as the main threat to the nation’s vitality in *Hygeia* (1803), Lane linked national vitality with social life: ‘You have only to look at the American, indeed, and you read it at a glance. Tall, sallow, sunken-cheeked, he has already quite lost the robust British type’.^159^

Furthermore, inspired by Andrew Combe’s ‘liberal vital physiology’,^160^ the restoration of a hygienic way of life was of paramount philosophical importance in Lane’s theory. Lane contended that city lifestyles, characterised by unrelenting work without rest, would inevitably lead to disease, the form of which would depend on an individual’s innate vitality: ‘The man’s capacity for continuing this galley-slave life will depend wholly on the amount of his constitutional powers, the stock-in-trade with which he works; and in exact proportion to these, one man will hold out longer than his neighbour’.^161^ This viewpoint partially aligned with the constitutional medicine of the time. Physicians tended to explain illness through individual susceptibilities to conditions, as reflected in the well-known medical report on child labour in factories (1832): ‘The evil consequence will be in proportion to the youth of the person, his delicacy, or otherwise, the natural constitution, the length of time he is confined, and the confinement of air’.^162^

However, compared with contemporary physicians’ emphasis on environmental impacts, Lane placed greater emphasis on the role of habits and life patterns, implying that, without altering one’s way of living, individuals’ health prospects would parallel those of the exploited factory children:

He [the one who continues the galley-slave life] will be sure to try medicine first. It comes easiest and gives least trouble…so matters proceed from bad to worse…. Did it ever occur to that man or his advisers, that, in the first place, it was utterly hopeless to think of curing his disease…while the manner of life in which lay the radical cause of all the mischief was still persevered in?^163^

For Lane, the most important task of ‘that man or his advisers’ is to identify how the laws of health were broken in one’s life, rather than focusing on reducing pain. He posited that bodily functions were naturally oriented towards health, a principle he believed was underscored by physiology. In his account of ‘fever and pain’, he described them as the bodily organs’ ‘efforts to protect themselves by throwing their irritation on the external nerves’.^164^ Although this account might be reminiscent of Sydenham’s Hippocratic approach to fevers, Lane’s perspective was distinctly based on deductive reasoning, unlike Sydenham’s empirical approach. Lane applied this concept of the body’s innate orientation not only to health but also to disease as a fundamental basis for a coherent understanding of both health and disease. This perspective resonated with John Forbes’s assertion that ‘diseases are truly natural though not normal conditions of the living animal body’, and ‘attention to this fact…constitutes one of the strongest a priori grounds for admitting the reasonableness and probability of the Natural Cure of diseases’.^165^ For Lane, the goal of restoring a hygienic lifestyle was not to impede Nature’s efforts to maintain its optimal state. Interventions aimed merely at alleviating symptoms could hinder this process. In this context, physiology provided crucial insights into the body’s ideal state.

Regarding Lane’s efforts to reconcile Romantic vitalism with laboratory medicine, this emphasis on physiology over pathology marks a departure from Bennett’s approach, although Bennett was also interested in reconciling ‘the traditional physiological discourse on the laws of health’ with academic physiology and laboratory science.^166^ Like Lane, Bennett critiqued contemporary medicine’s reliance on empirical methods, stating that ‘experience now, as in the infancy of the art, is the only guide’.^167^ As observed in his major work *Clinical Lectures on Principles and Practices of Medicine* (1856), Bennett frequently referenced ‘vitality’ and emphasised ‘hygienic regulations’, underscoring the natural healing mechanism of the body.^168^ Indeed, the gap between laboratory and Romantic medicine in the nineteenth century was not as pronounced, especially in Germany, where, despite the loud criticisms of the ‘speculative excesses of *Naturphilosophie*’,^169^ laboratory experimental medicine aligned more with Romantic than materialist philosophies.^170^

However, Bennett did not support Romantic conceptions such as the universality of disease or the crucial interrelationship between vitality and hygiene. While acknowledging that ‘pathology is only the physiology of disease,’ Bennett stressed the importance of pathology for accurate diagnosis.^171^ As an early advocate for using microscopy in diagnosing cancers, which he deemed incurable,^172^ he believed that ‘the curability or incurability of diseases, can only be determined by pathology’.^173^ By 1858, his histopathological disease explanations had overshadowed Alison’s constitutional disease theories at Edinburgh Medical School.^174^ While criticising physicians who ‘leave everything to nature’ in scepticism towards drug efficacy, Bennett emphasised ‘an earnest effort…to establish the science of medicine upon something like a solid foundation’.^175^ He argued that this required an understanding of ‘our position’ and a separation of ‘what is known from what is unknown’.^176^

Conversely, as indicated by his emphasis on comprehensive physiology over specific pathology, Lane was more concerned with explaining the ‘unknown’ realm – the enigmatic nexus between nature and cure. It was through his Romantic view of nature, combined with his naturalist vision of hygiene and vitalist interpretation of disease that he sought an ultimate answer to the question, ‘What is Medicine?’

## Conclusion

Defining ‘nature cure philosophy’ is notoriously difficult for historians, who often approached it in a ‘doxographical rather than historical’ manner, as observed in earlier studies by authors practising naturopathy (ND) or other nonconventional medicines.^177^ The challenge in exploring Victorian nature cure philosophy lies not in its being an uncharted territory in British medical and intellectual history but in the necessity of approaching it with utmost caution. While charting an ‘untrodden path’ within its historiography, this essay focused on Lane’s medical theories, which represented intellectual endeavours to revive the philosophy in the second half of the nineteenth century. Due to its theoretical focus, topics such as Lane’s advertisements, media responses to his works and his ‘hydropathic sanatorium’ practice are left for future research, possibly within the context of challenges faced by naturopathic clinicians in the Victorian medical market.

The establishment of naturopathy in the early twentieth century was deeply related to the public’s fascination with the medical undercurrent of nature cure during the latter half of the previous century. Despite its growth during the Victorian era, interest in nature cure among medical professionals declined sharply later in the century, with few venturing new theories. This period marked the last era where the emerging biomedical paradigm and popular nature cure philosophy coexisted within the medical profession’s minds. Lane’s nature cure philosophy, from the laws of health to the definitions of disease and medicine, subtly diverged from his era’s naturopathic trends, which inclined towards techno-centric perspectives and emphasised physical and spiritual purity. His Romantic naturalist interpretations of contemporary naturopathic notions, such as the ‘healing power of nature’, ‘laws of health’, and ‘vital force’, which he viewed as firmly grounded in ‘modern physiology’, were integrated into his theoretical framework. This supported his emphasis on the systematic application of ‘natural agencies’ and patients’ own efforts for health. However, during the ‘rebirth of physiology’, a period heralding the end of philosophical idealism,^178^ the chasm between Lane and contemporary pathology reflected the broader challenges Victorian nature cure philosophy encountered in late nineteenth-century medical discourse.

Lane’s nature cure philosophy was focused on the ‘unknowns’ in medicine’s relation to nature. This embrace of obscurity regarding the force of nature in developing scientific theses, a hallmark of Romantic naturalism, is also apparent in Chambers’ *Vestiges*, castigated as ‘anti-scientific’ in intellectual discourse for its lack of practical research, reliance on second-hand knowledge and disregard for proper scientific methods.^179^ Indeed, idealistic assumptions of medicine, such as Lane’s hygienic medicine, were marginalised by the growing reductionist approach, which catalysed the development of modern biomedicine. While the notion of the ‘laws of health’ retained social popularity into the next century, its meaning shifted to a set of practical public hygiene guides,^180^ devoid of the Romantic or holistic elements present in Lane’s medical theory. An article in *Science* (1911) labelled ‘hygienic medicine’ as an obsolete and unprofessional mode, describing it as ‘methods useful in keeping the body in healthful activity’ in the previous era, in contrast to ‘physiological medicine’ or ‘etiological medicine’, deemed more modern and scientific by the author.^181^

Nonetheless, it is meaningful to explore the intellectual dilemmas faced by Victorian physicians regarding the interplay between medicine and nature. Lane’s emphatic rejection of medical scepticism, combined with his Romantic appreciation for nature, succinctly captures and embodies his perspective on medicine and disease. While acknowledging medicine’s limitations, Lane sought to redefine physicians’ roles. However, it is far from this study’s aim to claim his historical significance. His medical theory, which aimed to reconcile medicine with nature and vitalism with laboratory medicine, failed to achieve its goals – a feat still unaccomplished in medical history and deemed improbable by today’s standards. Today’s medical experts do not speak of ‘Romanticism’, and in modern medicine, there is little room for such philosophical concepts.

However, the historical meaning of the Romantic philosophy of nature does not lie in proposing an alternative science but in offering ‘a noteworthy ontology of nature’.^182^ This perspective is especially poignant considering that, as Susanna Lindberg indicates, today’s common techno-scientific views on nature lack the capability of articulating their own existential implications.^183^ Lane’s intellectual pursuits in such a tumultuous era, questioning ‘What is medicine?’ and ‘What is disease?’, prompt a re-evaluation of how we conceptualise disease and medicine today. It is always daunting for clinicians, whether in the Victorian era or today, to question the foundational assumptions underpinning their practices. It is the susceptibility to subtle philosophical tensions that encourages clinicians to critically review themselves and step outside their comfort zones. This is why this historical essay explored the philosophical tensions between medicine and nature, stirred by Romanticism.

## Footnotes

1

F.M. Lehmann , ‘Nature Cure’, BMJ, 1, 4759 (1952), 653–4: 654; Richard H. Shryock , ‘Sylvester Graham and the Popular Health Movement, 1830–1870’, The Mississippi Valley Historical Review, 18, 2 (1931), 172–83: 175.

2

Hans A. Baer , ‘The Work of Andrew Weil and Deepak Chopra – Two Holistic Health/New Age Gurus: A Critique of the Holistic Health/New Age Movements’, Medical Anthropology Quarterly, 17, 2 (2003), 233–5012846118 10.1525/maq.2003.17.2.233: 234.

3

Julius A. Roth , Health Purifiers and Their Enemies: A Study of the Natural Health Movement in the United States with a Comparison to Its Counterpart in Germany (New York: Prodist, 1976).

4

Anonymous , ‘The Natural Treatment of Disease’, Chambers’s Journal of Popular literature, Science and Arts, 8, 191 (1857), 135–7: 135.

5
*Ibid.*, 137.

6

Kenneth D. Keele , *‘Clinical Medicine in the 1860s’, in* Medicine and Science in the 1860s: proceedings of the Sixth British Congress on the History of Medicine, University of Sussex, 6–9 September 1967 (London: Wellcome Institute of the History of Medicine,1968), 1–11 : 1.

7

M. Maurice Raynaud , ‘An Address on Scepticism in Medicine’, BMJ, 2, 1076 (1881), 268–73PMC226408720749969: 270.

8 His father, Elisha Lane, remained in Montreal, running a trading business as a partner of a company called Gibb & Lane, which imported liquor and groceries. Pierre Poulin , ‘Gibb, James’, in Dictionary of Canadian Biography, Vol. 8 (Toronto: University of Toronto Press, 1985), 2003.

9

Andrew Wear , ‘The History of Personal Hygiene’, in W.F. Bynum and R. Porter (eds.), Companion Encyclopedia of the History of Medicine (London: Routledge, 1993), 1283–308; Logie Barrow , ‘Why were Most Medical Heretics at Their Most Confident around the 1840s?’, in A. Wear and R. French (eds.), British Medicine in an Age of Reform (London: Routledge, 1991), 165–85; Virginia Smith , Clean: A History of Personal Hygiene and Purity (Oxford: Oxford University Press, 2007).

10

Virginia Smith , ‘Physical Puritanism and Sanitary Science: Material and Immaterial Beliefs in Popular Physiology, 1650–1840’, in W.F. Bynum and R. Porter (eds), Medical Fringe & Medical Orthodoxy (London: Croom Helm, 1987), 174–97; Charles E. Rosenberg , Right Living: An Anglo-American Tradition of Self-Help Medicine and Hygiene (Baltimore, MD: Johns Hopkins University Press, 2003).

11

Vladimir Jankovic , ‘The Last Resort: A British Perspective on the Medical South, 1815–1870’, Journal of Intercultural Studies, 27, 3 (2006), 271–98: 273.

12

Min Bae , ‘Locating Hygienic Medicine within the Intellectual History of Hygiene: Cases of E. W. Lane and T. R. Allinson’, *History and Philosophy of the Life Sciences* , 44, 4 (2022), 1–25 10.1007/s40656-022-00529-436282398.

13

Robin Price , ‘Hydropathy in England 1840–70’, Medical History, 25, 3 (1981), 269–807022064 10.1017/s002572730003458xPMC1139039: 269.

14
*Ibid.*, 280.

15

James Bradley and Marguerite Dupree , ‘Opportunity on the Edge of Orthodoxy: Medically Qualified Hydropathists in the Era of Reform, 1840–60’, Social History of Medicine, 14, 3 (2001), 417–3711811187 10.1093/shm/14.3.417: 421.

16

Bradley and Dupree, ‘A Shadow of Orthodoxy? An Epistemology of British Hydropathy, 1840–1858’, Medical history, 47, 2 (2003), 173–9412754763 10.1017/s0025727300056702PMC1044596; Bradley, Dupree , and Alastair Durie , ‘Taking the Water-Cure: The Hydropathic Movement in Scotland, 1840–1940’, Business and Economic History, 26, 2 (1997), 426–37.

17 Literature on the early history of naturopathy encompasses perspectives from both naturopathists and historians. The former is often more biographical than professionally historical, notably Iva Lloyd , The History of Naturopathic Medicine: A Canadian Perspective (Toronto: McArthur & Company, 2009); Friedhelm Kirchfeld and Wade Boyle , Nature Doctors: Pioneers in Naturopathic Medicine (Portland, OR: Medicina Biológica, 1994). The latter includes studies on the British scene, such as P.S. Brown , ‘Nineteenth-Century American Health Reformers and the Early Nature Cure Movement in Britain’, Medical History, 32, 2 (1988), 174–943287059 10.1017/s0025727300047980PMC1139856; Jane M. Adams , Healing with Water: English Spas and the Water Cure, 1840–1960 (Manchester: Manchester University Press, 2015), 153–83; Adams, ‘Developing Naturopathy in Interwar Britain’, in Nicola K. Gale and Jean V. McHale (eds), Routledge Handbook of Complementary and Alternative Medicine (London: Routledge, 2015), 63–73 .

18

P.S. Brown , ‘Medically Qualified Naturopaths and the General Medical Council’, Medical History, 35, 1 (1991), 50–77 2008122 10.1017/s0025727300053126PMC1036269.

19 Smith, *op. cit.* (note 9), 302.

20 Adams, *Healing with Water, op. cit.* (note 17), 154. The highly eclectic system, officially named ‘naturopathy’ by Benedict Lust (1872–1945), adopted Sebastian Kneipp’s (1821–1897) version of hydropathy, the second wave of hydropathy in late nineteenth-century Europe. In Britain, naturopathy was not incorporated into the national public health system as it was in Germany, nor did it actively seek its own way independently of mainstream medicine as it did in the US. See James C. Whorton , ‘Benedict Lust, Naturopathy, and the Theory of Therapeutic Universalism’, Iron Game History, 8, 2 (2003), 22–9; Thomas W. Maretzki and Eduard Seidler , ‘Biomedicine and Naturopathic Healing in West Germany: A Historical and Ethnomedical View of a Stormy Relationship’, Culture, Medicine and Psychiatry, 9, 4 (1985), 383–421 3907981 10.1007/BF00049231.

21

Margaret Pelling , ‘Contagion/Germ Theory/Specificity’, in W.F. Bynum and R. Porter (eds), Companion Encyclopedia of the History of Medicine (London: Routledge, 1993), 309–34: 325.

22

James C. Whorton , Nature Cures: The History of Alternative Medicine in America (Oxford: Oxford University Press, 2002); Susan E. Cayleff , Nature’s Path: A History of Naturopathic Healing in America (Baltimore, MD: Johns Hopkins University Press, 2016).

23 Whorton, *op. cit.* (note 22).

24 Brown, *op. cit.* (note 18); Bae, *op. cit.* (note 12).

25

Tomo Sato , ‘E. W. Lane’s Hydropathic Establishment at Moor Park’, Hitotsubashi Journal of Social Studies, 10, 1 (1978), 45–59 ; Gowan Dawson , Darwin, Literature and Victorian Respectability (Cambridge: Cambridge University Press, 2007). The first author’s name, 佐藤 共子 (Tomoko Sato), was wrongly published in English.

26

Kate Summerscale , Mrs Robinson’s Disgrace: The Private Diary of a Victorian Lady (London: Bloomsbury Publishing, 2012); Janice M. Allan , ‘Mrs Robinson’s “Day-Book of Iniquity”: Reading Bodies of/and Evidence in the Context of the 1858 Medical Reform Act’, in G. Depledge and A. Mangham (eds), The Female Body in Medicine and Literature (Liverpool: Liverpool University Press, 2010); Jr. Ralph Colp , ‘Charles Darwin, Dr. Edward Lane, and the “Singular Trial” of Robinson V. Robinson and Lane’, Journal of the History of Medicine and Allied Sciences, 36, 2 (1981), 205–137012232 10.1093/jhmas/xxxvi.2.205.

27

Hans A. Baer , ‘The Potential Rejuvenation of American Naturopathy as a Consequence of the Holistic Health Movement’, Medical Anthropology, 13, 4 (1992), 369–831545694 10.1080/01459740.1992.9966058; Maretzki and Seidler, *op. cit.* (note 20); Cayleff, *op. cit.* (note 22).

28 Whorton, *op. cit.* (note 22), 25.

29

Alice A. Kuzniar , The Birth of Homeopathy out of the Spirit of Romanticism (Toronto: University of Toronto Press, 2017).

30

Roderick D. Buchanan and James Bradley , ‘“Darwin’s Delay”: A Reassessment of the Evidence’, Isis, 108, 3 (2017), 529–52: 539.

31

Richard Yeo , ‘Science and Intellectual Authority in Mid-Nineteenth-Century Britain: Robert Chambers and “Vestiges of the Natural History of Creation”’, Victorian Studies, 28, 1 (1984), 5–31 .

32

Steven Frye , ‘Cormac McCarthy’s “World in Its Making”: Romantic Naturalism in the Crossing’, Studies in American Naturalism, 2, 1 (2007), 46–65 : 48.

33

Peter Dear , ‘Romanticism and Victorian Scientific Naturalism’, European Romantic Review, 26, 3 (2015), 329–40.

34 Summerscale, *op. cit.* (note 26). The novel is Dinah Maria Mulock , ‘The Water Cure’, in Nothing New. Tales, Vol. 2 (London: Hurst and Blackett, 1857).

35 Price, *op. cit.* (note 13), 279.

36 Bradley and Dupree, *op. cit.* (note 15).

37 Edward W. Lane, *Disput. Jur. Ad Lib. Xxvi Tit. 6 Digest.: Qui Petunt Tutores, Et Curatores, Et Ubi Petantur* (Edinburgh: publisher not identified, 1847).

38

Steven Shapin , ‘“Nibbling at the Teats of Science”: Edinburgh and the Diffusion of Science in the 1830s’, in I. Inkster and J. Morrell (eds), Metropolis and Province: Science in British Culture, 1780–1850 (Philadelphia, PA: University of Pennsylvania Press, 1983), 151–78.

39 For more details about George Drysdale, see J. Miriam Benn , The Predicaments of Love (London: Pluto Press, 1992); Michael Mason , The Making of Victorian Sexual Attitudes (New York: Oxford University Press, 1994); Tomoko Sato , ‘George and Charles Drysdale in Edinburgh’, (Reprinted from) Journal of Tsuda College, 12 (1980), 51–89 .

40

George R. Drysdale , The Elements of Social Science, 4th edn (London: E. Truelove, 1861), 335 .

41 Benn, *op. cit.* (note 39), 84.

42

Edward. W. Lane , Hydropathy: or, Hygienic Medicine (London: John Churchill, 1859), 146 .

43 Benn, *op. cit. (note 39)*, 84.

44

K. Codell Carter , The Decline of Therapeutic Bloodletting and the Collapse of Traditional Medicine (New Brunswick, NJ: Transaction Publishers, 2012), 117 .

45

Anonymous , ‘General Considerations on Epidemic Diseases’, Chambers’s Edinburgh Journal, 12 (1849), 132–6.

46

D.H.A. Boyd , ‘William Henderson (1810–72) and Homeopathy in Edinburgh’, Journal of the Royal College of Physicians of Edinburgh, 36 (2006), 170–817153153 : 176; Marguerite Dupree , ‘Making Medicine Scientific: James Young Simpson and the Controversy over Homoeopathy in Edinburgh’, in R. Mander and A. Nuttall (eds), James Young Simpson: Lad O Pairts (Erskine: Scottish History Press, 2011), 97–116 .

47 The name ‘Edward Lane’ appears on the Clinical Medicine Class List (December 1851–April 1852), Special Collections, Edinburgh University Library, LHB1.

48

Anonymous , ‘Obituary (John Hughes Bennett, M.D., F.R.S.E.)’, BMJ, 2, 771 (1875), 473–78: 475.

49

Terrie M. Romano , Making Medicine Scientific: John Burdon Sanderson and the Culture of Victorian Science (Baltimore, MD: Johns Hopkins University Press, 2002), 19 ; John H. Warner , ‘Therapeutic Explanation and the Edinburgh Bloodletting Controversy: Two Perspectives on the Medical Meaning of Science in the Mid-Nineteenth Century’, Medical History, 24, 3 (1980), 241–586997652 10.1017/s0025727300040308PMC1082653.

50

George Ballingall , Observations on the Site and Construction of Hospitals (Edinburgh: Maclachlan & Stewart, 1851).

51

Edward. W. Lane , Thesis: Notes on Medical Subjects, Comprising Remarks on the Constitutions and Management of British Hospitals (Edinburgh: James Hogg, 1853).

52 John James Drysdale (1815–92) was his wife’s stepbrother and a prominent Liverpool homeopath (co-editor of the *British Journal of Homeopathy* from 1846).

53

Phillip A. Nicholls , Homoeopathy and the Medical Profession (Beckenham: Croom Helm, 1988); Price, *op. cit.* (note 13).

54 One of the first advertisements for his establishment at Moor Park appears in *The Morning Post* (15 May 1854).

55 As the third wife of her late husband, a leading Tory member of the Edinburgh Town Council, she was pivotal in Lane’s relocation. Her philanthropic reputation in Edinburgh and financial backing were indispensable for Lane’s ventures in England.

56 He died in Lane’s care at the Moor Park establishment (on 14 August 1858).

57 Robert Chambers was Lane’s close friend connected to the Drysdale family. Lane published articles in *Chambers’s Edinburgh Journal* (1852) and *Chambers’s Encyclopaedia* (1863).

58 George Combe, ‘29 August 1856,’ *George Combe Diary* (National Library of Scotland, MS 7431, 1856).

59 Lane, *op. cit.* (note 42), 19.

60 Anonymous, ‘Hydropathy and Nature’, *The Morning Post*, 18 August 1859, 2.

61
*Ibid.*

62 Sudbrook Park was advertised around 260 times in major publications from 1860 to 1863, while Moor Park had approximately 120 advertisements from 1854 to 1860 in similar outlets.

63 Lane, *op. cit.* (note 42), 33.

64
*Ibid.*, v.

65
*Ibid.*, viii.

66 Between 1857 and 1869, the first and second editions of Lane’s book were advertised at least 69 times (16 times and 43 times for the first and second editions, respectively) across various publications, with *The Times* and *The Athenaeum* being the main platforms.

67

Edward. W. Lane , ‘What is Disease?’, The Medical Press and Circular, 68 (1874), 543–4: 544.

68

Edward. W. Lane , ‘What is Medicine?’, The Medical Press and Circular, 70 (1875), 6–7 : 6.

69

James H. Pickford , Hygiene or Health as Depending Upon the Conditions of the Atmosphere, Foods and Drinks, Motion and Rest, Sleep and Wakefulness (London: John Churchill, 1858); Lionel J. Beale , On Personal and Domestic Hygiene (London: John Churchill, 1855); Stephen H. Ward , The Science of Health (London: Society for Promoting Christian Knowledge, 1853).

70

Christopher Hamlin , ‘Public Health’, in M. Jackson (ed.), The Oxford Handbook of the History of Medicine (Oxford: Oxford University Press, 2011), 411–28.

71

Edward W. Lane , Old Medicine and New (London: J. & A. Churchill, 1873), 49 .

72

John Smedley , Practical Hydropathy (London: John Kendrick and W. Horsell, 1858), v.

73 Anonymous, *op. cit.* (note 4), 135.

74

James Wilson , The Principles and Practice of the Water Cure, 2nd edn (London: John Churchill, 1854), 3 .

75

Richard E. Morris , ‘The Victorian “Change of Air” as Medical and Social Construction’, Journal of Tourism History, 10, 1 (2018), 49–65 : 6.

76

R. Saundby , ‘Mechano-Therapeutics’, BMJ, 1, 2246 (1904), 161.

77 Adams, *Healing with Water, op. cit.* (note 17), 51.

78

Douglas P. Mackaman , Leisure Settings: Bourgeois Culture, Medicine, and the Spa in Modern France (Chicago: University of Chicago Press, 1998), 88 .

79

John Pemble , The Mediterranean Passion: Victorians and Edwardians in the South (Oxford: Clarendon Press, 1987). 92 .

80 Lane, *op. cit.* (note 71), 52.

81
*Ibid.*, 46.

82
*Ibid.*, 43.

83 In the preface to the second edition, Lane explained that he deleted the word ‘natural’ from the title to avoid giving the impression that he regarded ‘all other medical systems as un-natural.’ Lane, *op. cit.* (note 42), vii.

84
*Ibid.*, 11.

85

Gregory Claeys , Encyclopedia of Nineteenth Century Thought (London: Routledge, 2004), 52 .

86

John B. Halsted , Romanticism (London: Macmillan, 1969), 12 ; Iago Galdston , ‘The Romantic Period in Medicine’, Bulletin of the New York Academy of Medicine, 32, 5 (1956), 346–6213304557 PMC1805912: 359.

87 Lane, *op. cit.* (note 42), 157.

88

Roy Porter , Disease , Medicine and Society in England, 1550-1860 (Basingstoke, UK: Macmillan Education, 1987), 20 .

89 David Skae, ‘Acute mania, treatment’, *Lothian Health Services Archive* (Edinburgh University Library, GD16/1, 1854), 2.

90

Onno Oerlemans , Romanticism and the Materiality of Nature (Toronto: University of Toronto Press, 2004), 43 .

91

Humphry Davy , Consolations in Travel: Or, the Last Days of a Philosopher (London: John Murray, 1830), 173 PMC510776929919753.

92 Lane, *op. cit.* (note 42), 57.

93

Brittany Pladek , ‘“Soothing Thoughts”: Romantic Palliative Care and the Poetics of Relief’, European Romantic Review, 23, 3 (2012), 403–13.

94

Erwin H. Ackerknecht , Medicine at the Paris Hospital, 1794–1848 (Baltimore, MD: Johns Hopkins Press, 1967), 133 .

95

Max Neuburger , ‘An Historical Survey of the Concept of Nature from a Medical Viewpoint’, Isis, 35, 1 (1944), 16–28: 27 .

96

W.F. Wade , ‘Address in Medicine’, BMJ, 2, 1128 (1882), 251–5: 25110.1136/bmj.2.1128.251PMC226450620750269.

97

John H. Warner , The Therapeutic Perspective: Medical Practice, Knowledge, and Identity in America, 1820–1885 (Cambridge, MA: Harvard University Press, 1986), 191 .

98

Bennett Dowler , ‘Speculative and Practical Researches on the Supposed Duality Unity and Antagonism of Nature and Art in the Cure of Diseases’, New Orleans Medical and Surgical Journal, 15 (1858), 787–805 : 802.

99 Lane, *op. cit.* (note 42), 129.

100
*Ibid.*, 102–3.

101

Edward. W. Lane , ‘Home-Work for the Medical Missionary’, The Medical Press and Circular, 70 (1875), 71–3, 92–4: 72.

102 Lane described his establishment as ‘hydropathic sanatorium’ in his advertisements in the early 1860s.

103 Anonymous, *op. cit.* (note 4), 137.

104 Lane, *op. cit.* (note 42), 96.

105

Michael Worboys , ‘Practice and the Science of Medicine in the Nineteenth Century’, Isis, 102, 1 (2011), 109–1521667779 10.1086/658660; Carter, *op. cit.* (note 44).

106 Friedrich W.J. Schelling, *System des Transcendentalen Idealismus* (Tübingen, 1800), cited in Galdston, *op. cit.* (note 86), 361.

107 By the 1840s, European hygienists’ earlier focus on the ‘dynamic model of the body’, in which pain and pleasure were controversially interpreted as the body’s signs through which nature ‘speaks’ to the subject, shifted towards a stronger emphasis on self-discipline. Philipp Sarasin , ‘The Body as Medium: Nineteenth-Century European Hygiene Discourse’, Grey Room, 27 (2007), 48–65 : 59. For social and cultural perspectives regarding the rising moral concern of the Victorian era, see Frank Mort , Dangerous Sexualities: Medico-Moral Politics in England Since 1830 (London: Routledge, 2002).

108

James Bradley , ‘Medicine on the Margins? Hydropathy and Orthodoxy in Britain, 1840–60’, in W. Ernst (ed.), Plural Medicine, Tradition and Modernity, 1800–2000 (London: Routledge, 2002), 19–39 : 20.

109

Bartholomew Parr , The London Medical Dictionary (London: T. Davison, 1809), 520 .

110 He was the father of Lionel S. Beale (1828–1906), the eminent physician-microscopist and fervent supporter of the scientific approach to medicine as a professor at King’s College Hospital.

111 Beale, *op. cit.* (note 69), 17.

112

Alan Richardson , British Romanticism and the Science of the Mind (Cambridge: Cambridge University Press, 2001), 6.

113

James A. Secord , Victorian Sensation: The Extraordinary Publication, Reception, and Secret Authorship of Vestiges of the Natural History of Creation (London: University of Chicago Press, 2000).

114

George Combe , The Life and Correspondence of Andrew Combe (Edinburgh: Maclachlan and Stewart, 1850), 312 .

115 Lane, *op. cit.* (note 42), 68.

116

Peter J. Bowler and Iwan R. Morus , Making Modern Science: A Historical Survey (Chicago: University of Chicago Press, 2005), 176 .

117

Richard Holmes , The Age of Wonder: How the Romantic Generation Discovered the Beauty and Terror of Science (New York, NY: HarperCollins Publishers, 2009), xviii.

118

Bernard V. Lightman and Gowan Dawson , Victorian Scientific Naturalism: Community, Identity, Continuity (London: University of Chicago Press, 2014), 2–3 .

119 Lane, *op. cit.* (note 42), 149.

120

Heidi Rimke and Alan Hunt , ‘From Sinners to Degenerates: The Medicalization of Morality in the 19th Century’, History of the Human Sciences, 15, 1 (2002), 59–88 21038728 10.1177/0952695102015001073: 79.

121

Lionel J. Beale , The Laws of Health, in Relation to Mind and Body: A Series of Letters from an Old Practitioner to a Patient (London: John Churchill, 1851), 28 PMC1043179538210084.

122

Kelvin Rees , ‘Water as a Commodity: Hydropathy at Matlock’, in R. Cooter (ed.), Studies in the History of Alternative Medicine (Basingstoke, UK: Macmillan, 1988), 28–45 : 31.

123 Lane, *op. cit.* (note 42), 22, 24.

124
*Ibid.*, 25.

125
*Ibid.*, 27.

126
*Ibid.*, 37.

127

Guenter B. Risse , ‘Medicine in the Age of Enlightenment’, in Andrew Wear (ed.), Medicine in Society (Cambridge: Cambridge University Press, 1992), 149–96: 164.

128

William P. Alison , Outlines of Pathology and Practice of Medicine (Edinburgh: William Blackwood and Sons, 1844), 36 .

129 Pelling, *op. cit.* (note 21), 31.

130 Lane, *op. cit.* (note 67), 544.

131

Gavin Budge , Romanticism , Medicine and the Natural Supernatural: Transcendent Vision and Bodily Spectres, 1789–1852 (Basingstoke, UK: Palgrave Macmillan, 2012), 60 .

132 Lane, *op. cit.* (note 42), 103.

133

Susanna Lindberg , ‘The Remains of the Romantic Philosophy of Nature: Being as Life, or the Plurality of Living Beings?’, The New Centennial Review, 10, 3 (2010), 37–54 : 42.

134

Edward. W. Lane , Hygienic Medicine: The Teachings of Physiology and Common-Sense (London: J. & A. Churchill, 1885), 7 .

135

James R. Allard , Romanticism, Medicine, and the Poet’s Body (Farnham: Ashgate, 2007), 30 .

136

Russell T. Trall , The Hydropathic Encyclopedia: A System of Hydropathy and Hygiene, in Eight Parts, Vol. 1 (New York: Fowler and Wells, 1851), 30 .

137
*Ibid.*, 4.

138

James C. Whorton , Inner Hygiene: Constipation and the Pursuit of Health in Modern Society (Oxford: Oxford University Press, 2000), 18 10.1353/jsh.2002.003517078174.

139 Trall, *op. cit.* (note 136), 19.

140 Although Brown openly repudiated the notion of the healing power of nature, he argued that there was ‘only one form of treatment: the administration of stimulants.’ Guenter B. Risse , ‘Brunonian Therapeutics: New Wine in Old Bottles?’, Medical History, 32, S8 (1988), 46–62 : 48.

141

William P. Alison , ‘Observations on the Epidemic Fever Now Prevalent among the Lower Orders in Edinburgh’, Edinburgh Medical and Surgical Journal, 28, 93 (1827), 233–63PMC576685730330180.

142

Charles T. Wolfe and Motoichi Terada , ‘The Animal Economy as Object and Program in Montpellier Vitalism’, Science in Context, 21, 4 (2008), 537–7919245106 10.1017/s0269889708001956.

143 Hunter regarded the essential feature of the vital principle as self-preservation against putrefaction. See John Hunter , A Treatise on the Blood, Inflammation, and Gun-Shot Wounds (London: John Richardson, 1794), 107 .

144

Andrew Combe , The Principles of Physiology Applied to the Preservation of Health, and to the Improvement of Physical and Mental Education (Edinburgh: Adam & Charles Black, 1834), 190 PMC578107330330407.

145 Lane, *op. cit.* (note 42), 67.

146
*Ibid.*, 69.

147 Stanley Finger and Nicholas J. Wade, ‘The Neuroscience of Helmholtz and the Theories of Johannes Müller Part 1: Nerve Cell Structure, Vitalism, and the Nerve Impulse’, *Journal of the History of the Neurosciences*, 11, 2 (2002), 136–55: 140.

148 Pelling, *op. cit.* (note 21), 324.

149 Bradley and Dupree, *op. cit.* (note 16), 176.

150 Lane, *op. cit.* (note 42), 69.

151

Roy Porter , The Greatest Benefit to Mankind: A Medical History of Humanity (London: HarperCollins, 1997), 330–31.

152

William F. Bynum , Science and the Practice of Medicine in the Nineteenth Century (Cambridge: Cambridge University Press, 1994), 52–3.

153

Charles J.B. Williams , Principles of Medicine, Comprising General Pathology and Therapeutics, Etc (London: J. Churchill, 1843), 85–6.

154 Lane, *op. cit.* (note 42), 71–4.

155

Rick C. Looijen , Holism and Reductionism in Biology and Ecology (Dordrecht: Springer Science+Business Media, 2000), xiv–xv .

156

Stefano Poggi and Maurizio Bossi , Romanticism in Science: Science in Europe, 1790–1840 (London: Kluwer Academic Publishers, 1994), 158 .

157

Deborah Brunton , Medicine in Modern Britain 1780-1950 (London: Routledge, 2018), 33 .

158 Holmes, *op. cit.* (note 117), xix.

159 Lane, *op. cit.* (note 42), 60.

160 Smith, *op. cit.* (note 10), 188.

161 Lane, *op. cit.* (note 42), 51.

162

Charles Wing , Evils of the Factory System: Demonstrated by Parliamentary Evidence (London: Saunders and Otley, 1837), cxii .

163 Lane, *op. cit.* (note 42), 52.

164
*Ibid.*, 47.

165

John Forbes , *Of Nature and Art in the Cure of Disease*, 2nd ed. (London: John Churchill, 1857), 78 .

166

Elaine Thomson , ‘Physiology, Hygiene and the Entry of Women to the Medical Profession in Edinburgh C. 1869–c. 1900’, Studies in History and Philosophy of Science Part C: Studies in History and Philosophy of Biological and Biomedical Sciences, 32, 1 (2001), 105–26: 113.

167

John H. Bennett , ‘Contributions to Pathology and Rational Medicine: No.1(Introduction) How Should Medicine Be Advanced? With a Few Words in Reply to the Suggestions of Dr Forbes,’ Monthly Journal of Medical Science, 1, 1 (1846), 20–7: 21.

168

John H. Bennett , Clinical Lectures on the Principles and Practice of Medicine, 2nd edn (New York: Samuel S. and William Wood, 1858).

169

Andrew Cunningham and Nicholas Jardine , Romanticism and the Sciences (Cambridge: Cambridge University Press, 1990), 7.

170

Charles A. Culotta , ‘German Biophysics, Objective Knowledge, and Romanticism’, Historical Studies in the Physical Sciences 4 (1974), 3–38 : 4.

171 Bennett, *op. cit.* (note 167), 22.

172 Bennett, *On Cancerous and Cancroid Growths* (Edinburgh, Sutherland and Knox, 1849).

173 Bennett, *op. cit.* (note 167), 23.

174

Robert Christison , On the Changes in the Constitution of Fevers and Inflammations in Edinburgh, During the Last Forty Years (Edinburgh: Murray and Gibb, 1858), 1PMC530909329646658.

175 Bennett, *op. cit.* (note 168), 9.

176
*Ibid.*

177

Robert Jütte , ‘The Historiography of Nonconventional Medicine in Germany: A Concise Overview’, Medical History, 43, 3 (1999), 342–5810885128 10.1017/s002572730006539xPMC1044149: 344.

178

N. Westerhof , ‘A Short History of Physiology’, Acta Physiologica, 202, 4 (2011), 601–321722321 10.1111/j.1748-1716.2011.02286.x: 601.

179 Yeo, *op. cit.* (note 31), 5–6.

180

Charles Porter , School Hygiene and the Laws of Health: A Text-Book for Teachers and Students in Training (New York: Longmans, Green, 1908); Anonymous, ‘Instruction in Schools on the Laws of Health’, The Lancet, 164, 4235 (1904), 1231–2.

181

C.R. Bardeen , ‘Medicine and Sociology’, Science, 34, 877 (1911), 497–506 17795678 10.1126/science.34.877.497: 499.

182 Lindberg, *op. cit.* (note 133), 38.

183
*Ibid.*

